# Research nurses as practice facilitators to disseminate an asthma shared decision making intervention

**DOI:** 10.1186/s12912-020-00414-0

**Published:** 2020-05-18

**Authors:** Lindsay Shade, Kelly Reeves, Jennifer Rees, Lori Hendrickson, Jacqueline Halladay, Rowena J. Dolor, Paul Bray, Hazel Tapp

**Affiliations:** 1 Atrium Health, Department of Family Medicine Research, 2001 Vail Avenue, Suite 400B Mercy Medical Plaza, Charlotte, NC 28207 USA; 2 University of North Carolina at Chapel Hill, 160 N. Medical Drive, CB 7064, Chapel Hill, NC 27599 USA; 3 Duke University Medical Center, 2608 Erwin Road, Suite 210, Durham, NC 27705 USA; 4 Department of Family Medicine, University of North Carolina at Chapel Hill, 590 Manning Drive, CB 7595, Chapel Hill, NC 27599-7595 USA; 5 Division of General Internal Medicine, Department of Medicine, Duke University Medical Center, 200 Morris Street, 3rd Floor, Durham, NC 27701 USA; 6 Vidant Medical Group, 2000 Venture Tower Drive, Greenville, NC 27834 USA

**Keywords:** Practice facilitation, Dissemination, Implementation, Asthma, Shared decision making, Intervention, Nurse

## Abstract

**Background:**

Practice facilitation is a method of introducing and sustaining organizational change. It involves the use of skilled healthcare professionals called practice facilitators (PFs) to help address the challenges associated with implementing evidence-based guidelines and complex interventions into practice. PFs provide a framework for translating research into practice by building relationships, improving communication, fostering change, and sharing resources. Nurses are well positioned to serve as PFs for the implementation of complex interventions, however, there is little evidence currently available to describe nurses in this role. Additionally, the best strategies to implement complex interventions into practices are still not fully understood. Combining practice facilitation with the train-the-trainer model has the potential to spread knowledge and skills. Shared decision making (SDM), which involves patients and providers jointly engaging in decisions around treatment options, has been shown to improve outcomes for patients with asthma. The goal of this manuscript is to describe and evaluate the practice facilitation process from the ADAPT-NC Study which successfully utilized research nurses to implement a complex asthma SDM toolkit intervention into primary care practices.

**Methods:**

As part of a larger study, 10 primary care practices were recruited for a facilitator-led dissemination intervention involving a 12-week rollout of an asthma SDM toolkit (trial registration: 1.28.2014, #NCT02047929). An experienced lead PF trained research nurses as PFs from each of the 4 participating practice-based research networks (PBRNs) in a train-the-trainer model utilizing a one-day training event and subsequent remote meetings. Evaluation of PF engagement was measured through process improvement surveys.

**Results:**

Overall, the asthma SDM intervention was successfully implemented within the 4 PBRNs. All 10 facilitator-led practices remained engaged with their PFs, with 8 out of the 10 practices able to incorporate and sustain SDM visits or clinics. Responses from the surveys for process improvement yielded improved PF communication and team dynamics over time.

**Conclusions:**

This study demonstrated effective use of research nurses as practice facilitators during the dissemination of an asthma SDM intervention into primary care practices, adding to the knowledge of best practices by describing a model of large-scale implementation of a complex intervention through practice facilitation with nurses.

**Trial registration:**

“Comparing Traditional and Participatory Dissemination of a Shared Decision Making Intervention” was retrospectively registered at https://clinicaltrials.gov/ on January 28th, 2014 (NCT02047929).

## Background

Practice facilitation is a method of introducing and sustaining organizational change [1]. It involves the use of skilled healthcare professionals called practice facilitators (PFs) to help address the challenges associated with implementing evidence-based guidelines and complex interventions into practice [2]. PFs are individuals who work to achieve continuous quality improvement through a series of incremental plan-do-study-act cycles [1]. These PFs provide a framework for translating research into practice by building relationships, improving communication, fostering change, and sharing resources [3]. Throughout the literature, practice facilitation has been shown to improve evidence-based guideline adoption, preventive care, smoking cessation, chronic illness care including diabetes, and cancer care [2, 4–11].

Nurses are well positioned to serve as PFs for the implementation of complex interventions. During their training, nurses build skills around being detailed and organized, patient and resilient, and learn how to think critically to make quick decisions [12]. These attributes are essential for practice facilitation, however, there is little evidence currently available to describe nurses in this role.

Additionally, the best strategies to implement complex interventions into practices are still not fully understood. In healthcare, the train-the trainer model leverages the experience of one provider or clinician to train others, who in turn disseminate the information onto others in their workplaces or communities [13–16]. Combining practice facilitation with the train-the-trainer model has the potential to spread knowledge and skills to a greater extent. Utilizing research nurses as PFs in a train-the-trainer approach, we disseminated a complex intervention for asthma across the state of North Carolina.

Asthma is a complex and costly chronic lung disease that affects 1 in 13 Americans [17]. Over 24 million children and adults are living with asthma in the United States, annually accumulating 10.5 million office visits, 1.8 million emergency department (ED) visits, and 440,000 hospitalizations [18]. The burden of this health care utilization amounts to $56 billion per year in medical expenses, loss of productivity, and premature death [19]. Unfortunately, 10 Americans die every day from complications of their asthma [18]. Interventions are needed to improve patient outcomes given the high prevalence, morbidity, and mortality associated with asthma.

Improving provider adherence to guideline recommendations may help improve outcomes for patients with asthma [20, 21]. Providers often underutilize the National Hearts, Lung, and Blood Institute’s guidelines for managing asthma and adherence is thought to be poor in part because of guideline length and complexity [21–23]. The guideline’s stepwise approach for managing asthma involves medication selection in varying doses and combinations dependent upon the patient’s age, severity classification, and control level [22]. It can be challenging for busy providers to quickly process this multitude of information when making medication selections with an asthma patient. Interventions, such as shared decision making (SDM) that improve patient/provider communication and simplify the medication selection process may help improve guideline adherence and patients’ outcomes.

SDM is an approach to care delivery that involves patients and providers jointly engaging in decisions around treatment options [24]. In SDM, both the patient and provider share relevant information, thus partner in their health care decisions. For the patient, this may include their personal values and lifestyle choices; for the provider, this may include pertinent disease information and the benefits and risks of various medications. The patient and provider together express their preferences with regards to treatments, such as improving disease control, minimizing side effects, limiting costs, or prioritizing convenience of the regimen. The patient and provider then discuss several evidence-based options and work towards an agreement on a treatment regimen [25, 26]. This SDM process has been shown to improve medication adherence and clinically relevant disease outcomes for patients with asthma [26–29].

**Fig. 1:**
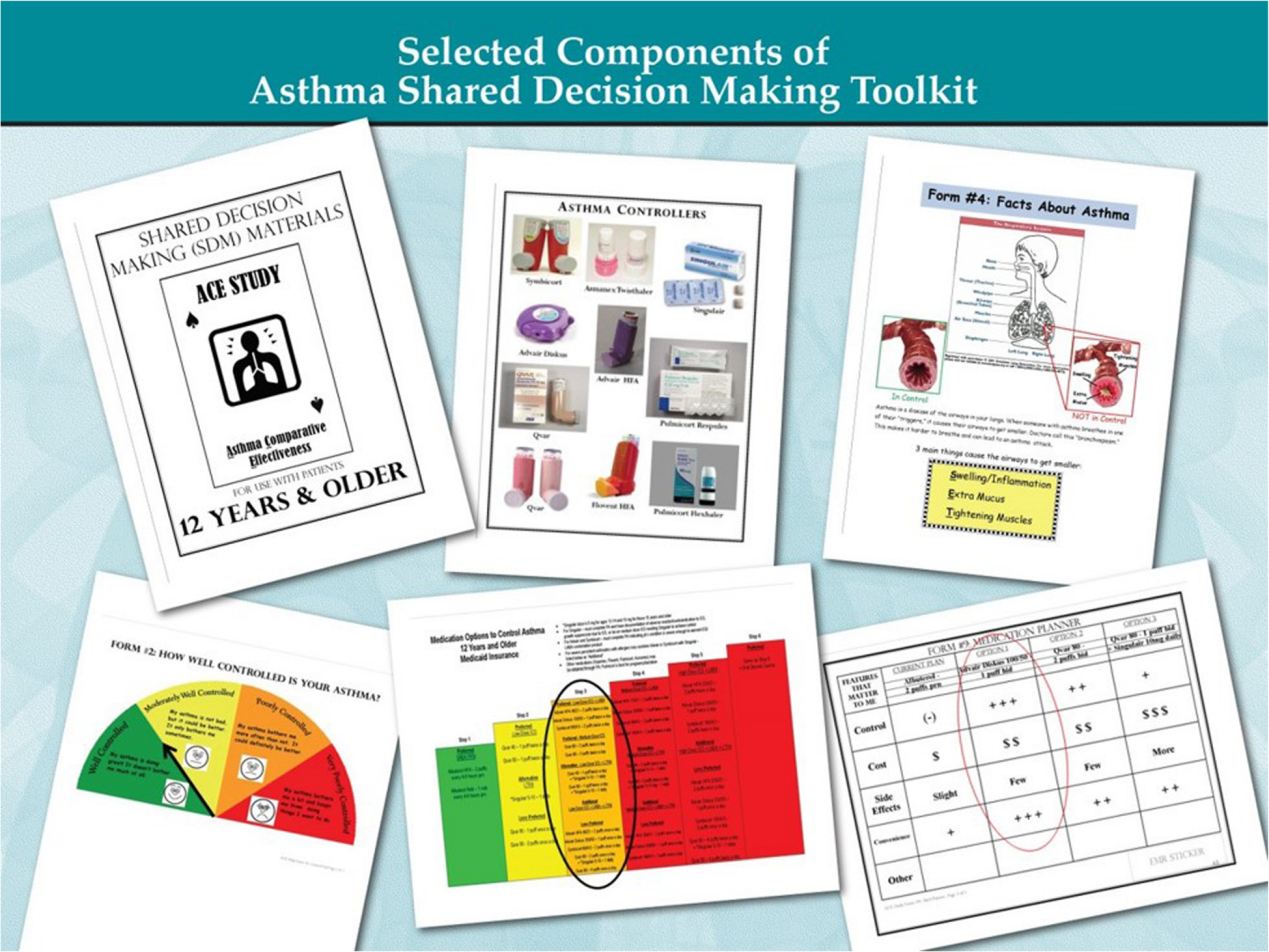
Selected Components of Asthma Shared Decision Making Toolkit. The images depicted in Fig. 1 are our own

North Carolina (NC) is home to over 635,000 patients with asthma, affecting 8.4% of its residents [30]. The Mecklenburg Area Partnership for Primary Care Research (MAPPR), a practice-based research network (PBRN) based in Charlotte, NC, previously developed and implemented an asthma SDM toolkit intervention at 6 underserved practices through practice facilitation [24, 27, 28, 31–34]. The intervention involved a team approach utilizing a health coach and toolkit comprised of decision aids, including evidence-based treatment options to streamline the medication selection process and improve asthma control (Fig. 1). To incorporate SDM for asthma care into the practices, the toolkit intervention focused on 5 essential components of SDM: (1) establishing the patient’s perception of current asthma control; (2) addressing medication adherence; (3) providing asthma education including “what is asthma,” controller versus rescue medications, correct inhaler technique, and trigger avoidance; (4) establishing treatment goals and medication preferences; (5) and finally negotiating several evidence-based treatment options based on the patient’s actual severity or control level. Using elements of the chronic care model and community-placed research, the complex toolkit intervention was tailored to fit the needs of each practice individually over a 12-week rollout period [27, 31, 35, 36]. Results from this pragmatic pilot study showed significantly reduced exacerbation rates in pediatric patients with asthma [27].

From 2013 to 2016, the ADAPT-NC (Asthma Dissemination Around Patient-centered Treatments in North Carolina) Study evaluated dissemination methods of the asthma SDM toolkit intervention through a large comparative effectiveness study, building on the work from our previous pilot [24, 27–29, 31–34, 37–42]. Research nurses were trained as PFs to disseminate the intervention. Results of the ADAPT-NC Study showed a significantly higher portion of asthma patients shared equally in the decision-making with their provider in the facilitator-led dissemination arm (75%; 95% CI [71.7, 78.1]) compared to a traditional lunch-and-learn approach (66%; 95% CI [62.8, 69.8]) (*p* = 0.001) [29]. The facilitator-led arm also had the lowest proportion of patients visiting the ED (14% decrease for facilitator-led (95% CI [+ 4%, − 32%], *p* = 0.21), 12% decrease for traditional lunch-and-learn (95% CI [+ 11%, − 35%], *p* = 0.09), and 9% increase for usual care (95% CI [+ 28%, − 10%], *p* = 0.28)) [29]. These results suggest that complex interventions such as SDM are most effectively implemented in practices using a structured, facilitator-led approach.

The goal of this manuscript is to describe and evaluate the practice facilitation process from the ADAPT-NC Study which successfully utilized research nurses to implement a complex asthma SDM toolkit intervention into primary care practices.

## Methods

### Setting

The ADAPT-NC Study leveraged the partnerships of 4 PBRNs to disseminate SDM for asthma care. The 4 PBRNs are: (1) MAPPR, the lead group, affiliated with Atrium Health (formerly Carolinas HealthCare System) in Charlotte; (2) North Carolina Network (NCnet) affiliated with the University of North Carolina at Chapel Hill; (3) Primary Care Research Consortium (PCRC) affiliated with Duke University in Durham; and (4) Eastern Carolina Association for Research and Education (E-CARE) affiliated with East Carolina University and Vidant Health System in Greenville.

As previously described [37], 30 primary care practices widely distributed across the state of North Carolina were recruited for the ADAPT-NC Study by the 4 PBRNs. Briefly, 476 primary care practices were eligible to participate by having at least 75 patients with a diagnosis of asthma and Medicaid insurance. The PBRNs recruited practices in their geographic regions on a voluntary basis through in-person conversations, email, and/or phone calls. MAPPR and NCnet each recruited 9 practices; PCRC and E-CARE each recruited 6 practices.

### Study design

**Fig. 2:**
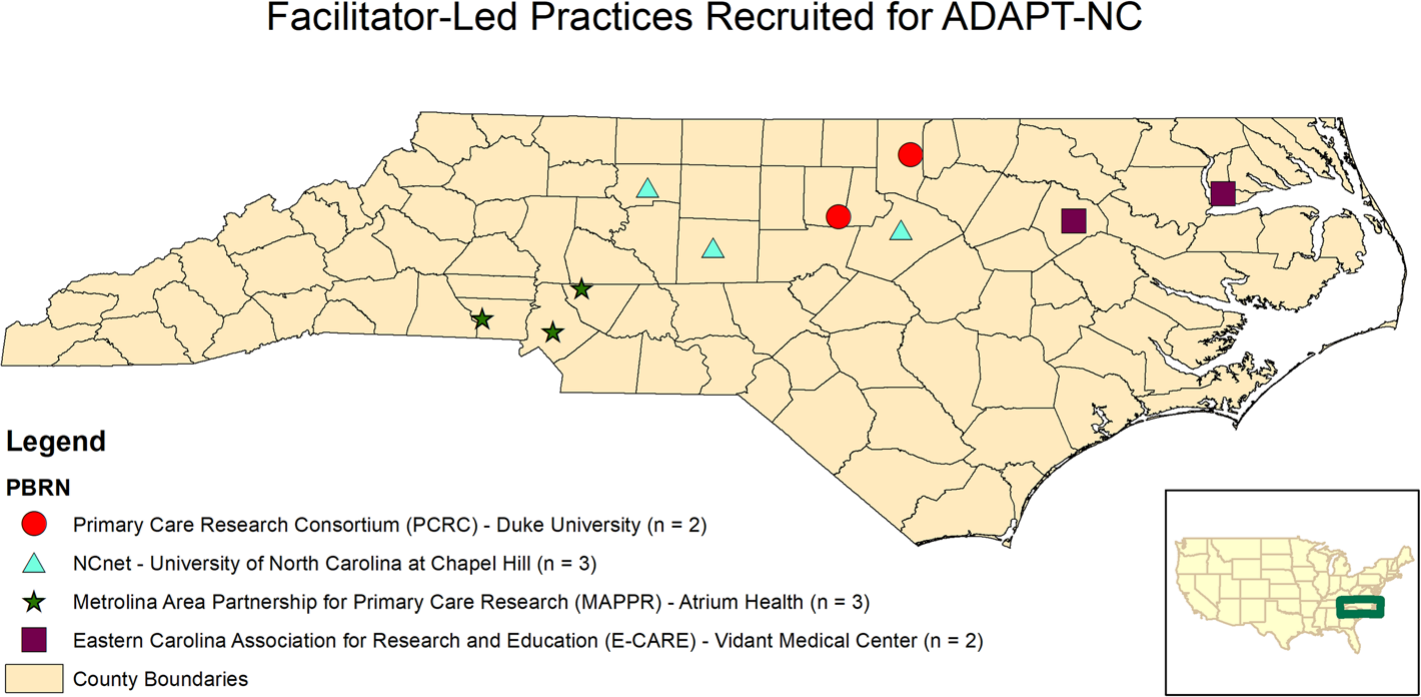
Geographic Distribution of Facilitator-Led Practices Recruited by Practice-Based Research Network across North Carolina. The map depicted in Fig. 2 is our own

The objective of the ADAPT-NC Study was to compare 3 dissemination strategies for implementing an asthma SDM toolkit intervention into primary care practices [29]. As previously described, 10 practices were cluster randomized into each of the 3 arms: (1) facilitator-led dissemination involving a 12-week rollout of the toolkit intervention; (2) traditional dissemination involving a one-hour lunch-and-learn presentation of the intervention; and (3) a usual care control group with no active intervention. This manuscript focuses on the 10 practices randomized into the facilitator-led arm of the ADAPT-NC Study (Fig. 2). Baseline practice level data is displayed in Table 1. Overall, the practices’ asthma patients were largely pediatric. Number of providers ranged from 2 to 34 with 6 practices part of a healthcare system and 4 private practices.

**Table 1:** Facilitator-Led Practices Baseline Data

PBRN	Site #	Practice Setting	# Providers	# Patients	# Asthma Patients (% Practice)	% Pediatric Asthma Patients
MAPPR	1	Healthcare System	2	690	78 (11.3)	55.1
	2	Healthcare System	6	4,326	401 (9.3)	100.0
	3	Healthcare System	9	2,909	244 (8.4)	100.0
NCnet	4	Private Practice	6	1,590	120 (7.5)	65.8
	5	Private Practice	34	2,950	375 (12.7)	100.0
	6	Private Practice	7	2,130	205 (9.6)	97.1
PCRC	7	Private Practice	9	2,529	352 (13.9)	99.7
	8	Healthcare System	8	1,384	156 (11.3)	77.6
E-CARE	9	Healthcare System	6	1,028	122 (11.9)	51.6
	10	Healthcare System	3	2,121	290 (13.7)	100.0
**Total Combined**			**90**	**21,657**	**2,343 (10.8)**	**84.7**

*PBRN* Practice-based research network, *MAPPR* Mecklenburg Area Partnership for Primary Care Research, *NCnet* North Carolina Network, *PCRC* Primary Care Research Consortium, *E-CARE* Eastern Carolina Association for Research and Education

### Practice facilitators

An experienced lead PF from MAPPR, a physician assistant, trained PFs from each of the 4 participating PBRNs in a train-the-trainer model. MAPPR, NCnet, and E-CARE each utilized 1 full-time PF whereas PCRC had 2 part-time PFs. The PFs were all registered nurses with bachelor’s degrees and had over 100 years of combined nursing experience. The PBRN PFs all had previous experience in research as well as health coaching and/or practice facilitation with additional certifications including Certified Practice Facilitator (CPF), Certified Clinical Research Professional (CCRP), and Certified in User Experience (UXC).

### Training day

A one-day training event was held for PBRN PFs and additional researchers during which the 12-week rollout of the asthma SDM toolkit intervention was explained in detail, with emphasis placed on using an adaptable and flexible approach to implementation. A manual of operating procedures was utilized to outline a general framework for the study, highlighting the aim to weave participation at the PBRN and practice level through every aspect of disseminating asthma SDM. Education included an asthma review, inhaler technique and peak flow meter teaching, and toolkit overview.

### Practice facilitator remote meetings

Following the training day, ongoing biweekly PF remote meetings with video conferencing and screen sharing capabilities were facilitated with the PFs who were located throughout the state. These online meetings allowed the PFs to learn the intervention from the lead PF, share best practices and lessons learned with each other, as well as problem solve challenges together. The lead PF presented topics such as additional asthma education, recruitment tips, step-by-step intervention training, and how to promote sustainability of the intervention. The PF remote meetings were recorded so topics could be revisited by the PFs as needed.

### Practice facilitator process improvement surveys

Evaluation of PF engagement was measured through process improvement surveys. One year after the rollouts began and again 6 months later, the PBRN PFs were sent anonymous surveys evaluating team dynamics and communication preferences to elicit improvement suggestions. The feedback was used by the lead PF to iteratively adapt to the needs of the group as the study progressed. Please see Supplementary File 1 for the complete Practice Facilitator Process Improvement Survey.

### Facilitator-led dissemination intervention

**Fig. 3:**
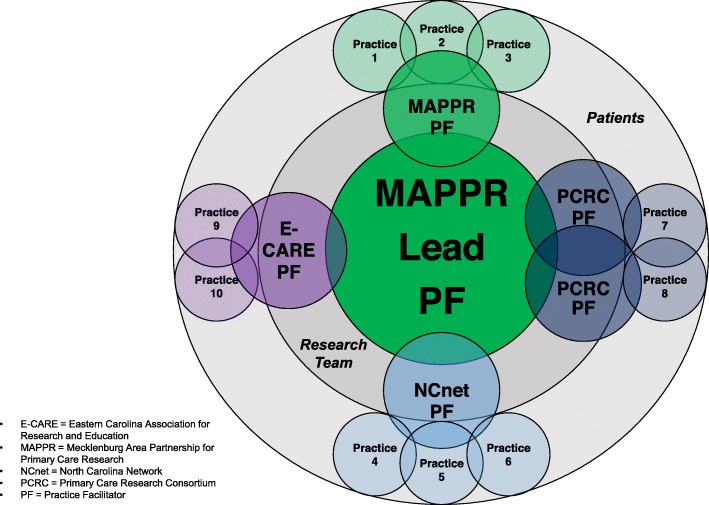
Facilitator-Led Dissemination Model of Practice Facilitation

Figure 3 depicts how the lead PF interacted with the PFs from the 4 PBRNs, supported by their research teams, at the 10 facilitator-led practices.

**Fig. 4:**
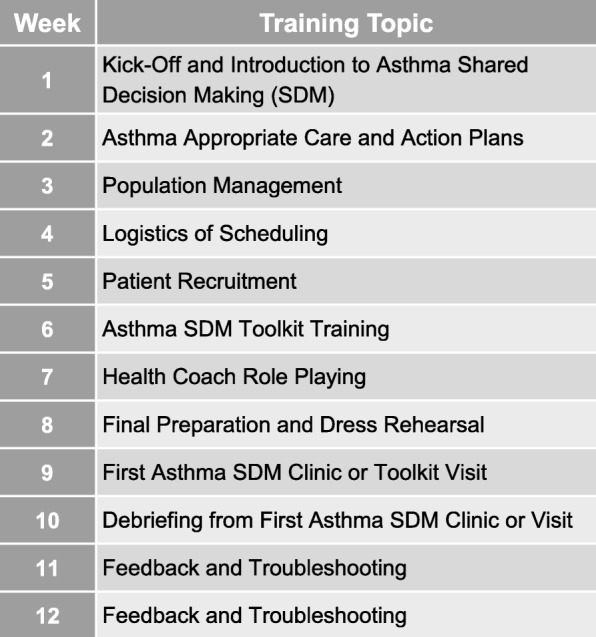
Facilitator-Led Intervention 12-Week Rollout Schedule

PFs from the PBRNs trained their facilitator-led practices in the asthma SDM intervention, adapting it to their practices’ cultures, over a 12-week rollout period. The 12-week rollouts consisted of weekly hour-long meetings at the practice’s convenience and included key personnel to form a core team typically comprised of a provider champion, practice manager, health coach, nurses, and registration staff [24, 37]. Each week the PF led the core team through a new training topic including: asthma appropriate care and action plans, population management, logistics of scheduling, patient recruitment, and asthma SDM toolkit training, all culminating in the development of asthma SDM visits or clinics at the practice’s discretion. The PF assisted the core team in adapting the toolkit from the previous pilot study [39] into a version that suited their practice’s specific needs. Time was allotted to allow the core team at the practice to role play the health coaching toolkit process and work through the visits or clinics in a dress rehearsal fashion. Generally, by week 9 of the rollout, the practice was encouraged to see their first asthma patients for SDM. The remaining weeks of the rollout involved debriefing, troubleshooting, and feedback to improve the process in preparation for future SDM visits or clinics (Fig. 4).

After 1 year, the PF revisited their facilitator-led practices and met again with their core teams for “refresher” training sessions, aiming to promote sustainability of the intervention. The refresher sessions were 1 h-long meetings where success was celebrated, barriers were tackled, and next steps such as adding additional providers and/or health coaches to the intervention’s core team were planned.

Throughout the 3-year project, the PFs were available to their facilitator-led practices for additional training or consultation as requested. Altogether the PFs spent a minimum of 13 h on site at each practice while hosting the training sessions during the rollouts and refreshers, plus an additional 1–4 h per week on average in the 18 months post rollout responding to various questions and concerns depending on the practice’s individual needs. Requests included, for example, assistance with additional asthma education for clinical staff or more health coaching practice with the toolkit. Therefore, the total dosage of PF support ranged from approximately 100–400 h throughout the entire study period.

### Facilitator-led monthly calls

Core team members from the 10 facilitator-led practices were invited to participate in a monthly call with the PFs and PBRN researchers in order to encourage collaboration, share lessons learned, brainstorm, and discuss relevant project updates. Each call centered on a theme to guide the discussion. Core team members from the facilitator-led practices were engaged to suggest call themes which included: patient recruitment, flu shots, SDM documentation, productivity and billing, maintenance of certification and patient centered medical homes, SDM clinic or visit scheduling, new staff training, and medication options to control asthma. The first call took place after all PBRNs had rolled out at their practices. After the 6th call, the calls moved to bimonthly in response to the group’s feedback then continued through the end of the study. A total of 15 calls were held altogether.

## Results

Overall, the asthma SDM intervention was successfully implemented within the 4 PBRNs. All 10 facilitator-led practices remained engaged with their PFs, receiving at least 100 h of PF support in the 18 months post rollout, with 8 out of the 10 practices able to incorporate and sustain SDM visits or clinics. Of the 2 practices not able to fully implement, 1 practice experienced a 75% staffing turnover within the first year of the project and chose to focus on day-to-day clinic operations instead of SDM for asthma care. The other practice struggled with provider buy-in secondary to lack of leadership support in prioritizing SDM.

As previously described, the facilitator-led practices reported higher levels of SDM occurring in their asthma visits compared to the traditional lunch-and-learn practices (75% vs 66%, *p* = 0.001) and the facilitator-led practices also had the lowest proportion of patients visiting the ED for exacerbations [29].

### Highlights and obstacles

Table 2 summarizes some of the highlights and obstacles that the PBRN PFs encountered. Each PF had the opportunity to work with a mix of practices, including one or more highly motivated practices that rose to the challenge of incorporating this complex chronic disease intervention into their daily routine.

**Table 2:** Practice Facilitation Highlights and Obstacles

PBRN	Site #	Highlights	Obstacles
MAPPR	1	Introduced the concept of SDM; provided basic asthma education to staff	Unable to adopt SDM due to competing priorities
	2	Transitioned from group shared medical appointments to individualized SDM; evolved to become a self-sufficient team	Resistant to change at first
	3	Eagerly engaged early on; willing to commit to team approach for SDM care	Provider, staff, and administrative turnover
NCnet	4	Completed everything asked and more; developed video to promote SDM for patients	Not all providers participated; long distance for PF to travel to practice
	5	All providers at 5 locations within group practice engaged; tech-savvy, modified SDM tools for their website	All 5 locations wanted to receive the intervention simultaneously; PF unable to visit all locations
	6	Provided basic asthma education to staff	Long distance for PF to travel to practice; provider and staff turnover
PCRC	7	PF process built relationships for future research opportunities; staff excited to take on new roles	Difficult to find suitable meeting time for providers and staff together; provider champion had competing priorities for time
	8	Enthusiastic team interested in new roles; staff growth; became more comfortable with SDM	Difficult to reach staff by phone and email; long distance for PF to travel to practice
E-CARE	9	Introduced different length visits to accommodate more SDM opportunities	Provider motivation; health coaching was a new concept at the practice; few referrals initially
	10	Developed reminder system to engage providers	Provider and staff turnover

*PBRN* Practice-based research network, *MAPPR* Mecklenburg Area Partnership for Primary Care Research, *NCnet* North Carolina Network, *PCRC* Primary Care Research Consortium, *E-CARE* Eastern Carolina Association for Research and Education, *SDM* Shared decision making

One practice filmed a video to promote SDM within their community. Another practice that was in a network with 5 locations requested to receive the intervention simultaneously which was achieved through deployment of a live conferencing system with cameras in meeting rooms at each location, allowing the PF to train the multi-site group together. A PF struggled with engagement at a practice after the provider champion shifted to working part-time until the solution of working more closely with the practice’s staff helped the momentum return.

Common obstacles included provider and staff turnover, lengthy distances for the PFs to travel to some of their practices, challenges finding a common meeting time for core team members and their PF, as well as difficulty reaching busy providers by phone and email.

### Practice facilitator process improvement surveys

Responses from the initial and follow-up PF surveys for process improvement yielded improved PF communication and team dynamics over time (Table 3). Compared with the initial survey, the follow-up survey indicated the PFs overall felt that there was more acknowledging and more contributions of ideas and opinions, as well as less speaking for another person, interrupting, questioning, and disagreeing. Other survey-related improvements included more direct communication, functioning as a team, cooperation, listening, respect, and ideas valued. The PFs indicated in follow-up that they felt the project was more on track with improved use of their time and skills. Midway through the intervention, the biweekly PF remote meetings were changed to monthly in response to the feedback.

**Table 3:** Practice Facilitator Process Improvement Survey Results: Response Averages from Initial and Follow-Up Surveys

**Question 1: Considering the dynamics of the team, how much do you think that each of the following behaviors occur when we meet and interact with each other?**		**Initial Survey** ***n*** **= 5** **mean (SD)**	**Follow-Up Survey** ***n*** **= 3** **mean (SD)**
Positive Attributes	Contributing ideas and opinions	3.2 (0.8)	4.0 (0.0)
	Acknowledging	3.4 (0.9)	4.0 (0.0)
	Agreeing	3.6 (0.9)	3.7 (0.6)
	Negotiating	3.2 (1.1)	3.7 (0.6)
	People speaking up for themselves and their opinions	3.3 (0.5)	3.0 (1.0)
	Summarizing	3.8 (0.8)	4.0 (1.0)
Negative Attributes	Disagreeing	2.2 (0.4)	1.7 (0.6)
	Interrupting	3.4 (0.5)	3.0 (0.0)
	Questioning	3.2 (0.8)	2.3 (0.6)
	Speaking for another person	2.6 (0.9)	1.7 (0.6)
**Question 2: Please select the response that represents the extent to which the following statements apply to the research team**		**Initial Survey** ***n*** **= 5** **mean (SD)**	**Follow-Up Survey** ***n*** **= 3** **mean (SD)**
Decisions made are being put into action		3.6 (0.9)	4.0 (1.0)
Everyone’s ideas were valued		3.4 (0.9)	4.0 (1.0)
I got enough information to understand the big picture		3.8 (0.8)	4.0 (1.0)
I was motivated to put forth my best efforts		3.8 (0.8)	4.0 (1.0)
I was respected		3.4 (1.5)	4.3 (0.6)
I was told when I did a good job		3.6 (1.1)	4.3 (1.2)
Our decisions stayed on track		3.6 (0.9)	4.0 (0.0)
People functioned as a team		3.5 (0.9)	4.0 (0.0)
People were cooperative and considerate		3.4 (0.9)	4.0 (0.0)
People were direct and honest with each other		3.4 (0.9)	4.0 (0.0)
People were good listeners		3.0 (0.7)	3.7 (0.6)
The meeting tapped the creative potential of all people present		2.8 (0.8)	3.7 (0.6)
Time was well spent		2.8 (0.8)	3.7 (0.6)

Answer choices: (1) to a very little extent; (2) to a little extent; (3) to some extent; (4) to a great extent; (5) to a very great extent

All 5 PFs completed the initial survey and 3 of the 5 PFs completed the follow-up survey

*SD* Standard Deviation

## Discussion

Practice facilitation utilizing research nurses brought a comprehensive approach to implementation of a SDM toolkit intervention for patients with asthma in the ADAPT-NC Study. Leveraging the train-the-trainer model and elements of community-placed research, this facilitator-led implementation demonstrates effective use of research nurses as PFs to disseminate SDM for asthma care into primary care practices offering the potential for broader dissemination of complex interventions utilizing remote meeting capabilities and research nurses as PFs.

This study adds to the developing knowledge of best practices for implementation of large-scale, complex, primary care interventions through practice facilitation. Baskerville et al. concluded in their systematic review that primary care practices are nearly 3 times more likely to adopt evidence-based guidelines through practice facilitation when there is tailoring of the intervention [7]. Qualitative studies of primary care practice facilitation of complex interventions echo the importance of facilitating team communication, iterative evaluation of the implementation process with real-time feedback, facilitator integration into the practices, and flexibility [43, 44].

To date, there is little evidence directly describing the role of nurses as practice facilitators. However, attributes of nurses most likely associated with success in practice facilitation are well described. These include their empathetic approaches to care, inter- and multidisciplinary team collaboration, as well as training in protocols, evidence-based care, quality improvement, leadership, and communication skills [45–50].

Research nurses demonstrated these skills during intervention rollouts in the ADAPT-NC Study. Not only had their training taught them to be empathetic and adaptable problem solvers, they also displayed versatile disease knowledge and understanding, along with effective communication skills. This model of practice facilitation leveraging nurses suggests potential as a method to implement research into practice, which may be further adapted to suit the needs of other populations, diseases or conditions, and settings [51].

Throughout the practice facilitation process in the ADAPT-NC Study, the PFs expressed common lessons learned: to be patient, persistent, pliable, and persevere. The PFs needed to patiently await responses from busy providers caring for patients first. Persistence in the form of frequent reminders was important for the PFs to keep the project running on track, sometimes arriving in person if the situation permitted. The PFs showed pliability when providing their practices with ample options and resources to tailor the intervention to their individual needs. Adapting to the changing needs of the varying practices required perseverance in the form of flexibility and quick thinking. The importance of establishing a trusting relationship with the practice, then building upon it with and frequent communication, was paramount to success.

### Limitations

In line with other real-world, pragmatic implementations, our study had several limitations. First, the 10 facilitator-led practices all agreed voluntarily to participate in the study, introducing a possible source of selection bias.

While most practices were highly motivated to integrate asthma SDM, each PBRN had 1 practice that struggled to meet the demands of this complex project. Frequent staff turnover and lack of provider buy-in proved to be the main limitations of struggling practices. It was challenging for the practices and PFs to continually train newly hired staff with no previous knowledge of the implementation or SDM intervention for asthma care. Staff motivation was impacted when practices lacked strong leadership such as a champion to support the program.

With the small number of PFs involved in this study and in completing process improvement surveys, there was a risk that social desirability and unmasking may have influenced their survey responses.

While we were able to provide process improvement data back to the PFs, due to delays in obtaining disease outcome data we were not able to supply the 10 facilitator-led practices with clinically relevant outcomes results as originally planned.

## Conclusions

This study demonstrated effective use of research nurses as practice facilitators during the dissemination of an asthma SDM intervention into primary care practices across the state of North Carolina, adding to the knowledge of best practices by describing a model of large-scale implementation of a complex intervention through practice facilitation with nurses. Future randomized controlled trials using practice facilitation should incorporate cost effectiveness analysis to determine the potential impacts of wider adoption.

## Supplementary information

**Additional file 1: Supplementary File 1.** Practice Facilitator Process Improvement Survey. Survey used to evaluate practice facilitator engagement, including team dynamics and communication preferences, to elicit improvement suggestions over time.

## Data Availability

All data generated and analyzed during this study are included in this published article.

## Abbreviations

ADAPT-NC: Asthma Dissemination Around Patient-centered Treatments in North Carolina
CCRP: Certified Clinical Research Professional
CPF: Certified Practice Facilitator
E-CARE: Eastern Carolina Association for Research and Education
ED: Emergency Department
MAPPR: Mecklenburg Area Partnership for Primary Care Research
NC: North Carolina
NCnet: North Carolina Network
PCORI: Patient-Centered Outcomes Research Institute
PBRN: Practice-based research network
PF: Practice facilitator
PCRC: Primary Care Research Consortium
SDM: Shared decision making
UXC: Certified in User Experience
