# Supplementary material for: Research nurses as practice facilitators to disseminate an asthma shared decision making intervention
Source: BMC Nurs. 2020 May 18;19:40. doi: 10.1186/s12912-020-00414-0 (PMC7236364; doi:10.1186/s12912-020-00414-0)
Supplement: Supplementary file 1 — Additional file 1: Supplementary File 1. Practice Facilitator Process Improvement Survey. Survey used to evaluate practice facilitator engagement, including team dynamics and communication preferences, to elicit improvement suggestions over time. [file 12912_2020_414_MOESM1_ESM.pdf]

Practice Facilitator Process Improvement Survey

Question 1: Considering the dynamics of the team, how much do you think that each of the following behaviors occur when we meet and interact with each other?

Answer choices: (1) to a very little extent; (2) to a little extent; (3) to some extent; (4) to a great extent; (5) to a very great extent

- Contributing ideas and opinions
- Acknowledging
- Agreeing
- Negotiating
- People speaking up for themselves and their opinions
- Summarizing
- Disagreeing
- Interrupting
- Questioning
- Speaking for another person

Question 2: Please select the response that represents the extent to which the following statements apply to the research team.

Answer choices: (1) to a very little extent; (2) to a little extent; (3) to some extent; (4) to a great extent; (5) to a very great extent

- Decisions made are being put into action
- Everyone's ideas were valued
- I got enough information to understand the big picture
- I was motivated to put forth my best efforts
- I was respected
- I was told when I did a good job
- Our decisions stayed on track
- People functioned as a team
- People were cooperative and considerate
- People were direct and honest with each other
- People were good listeners
- The meeting tapped the creative potential of all people present
- Time was well spent

Question 3: Do you have any suggestions about how the team could work better together?

Question 4: What do you think is the biggest barrier to our success?

Question 5: What do you like best about working on the team?
